# Supplementary material for: Investigation of Strategies to Block Downstream Effectors of AT1R-Mediated Signalling to Prevent Aneurysm Formation in Marfan Syndrome
Source: Int J Mol Sci. 2024 May 4;25(9):5025. doi: 10.3390/ijms25095025 (PMC11084825; doi:10.3390/ijms25095025)
Supplement: Supplementary file 1 [file ijms-25-05025-s001.zip › ijms-2942775-supplementary.pdf]

Investigation of strategies to block downstream effectors of AT1R mediated signalling to prevent aneurysm formation in Marfan Syndrome

Irene Valdivia Callejon[1,*], Lucia Buccioli [1,*], Jarl Bastianen[1], Jolien Schippers[1], Aline Verstraeten[1], Ilse Luyckx[1,3], Silke Peeters[1] , A.H. Jan Danser[3], Roland Van Kimmenade[4], Josephina Meester[1], Bart Loeys[1,3, **]

1. Centre of Medical Genetics, Antwerp University Hospital/University of Antwerp, Antwerp, Belgium;
2. Department of Human Genetics, Radboud University Medical Center, Nijmegen, The Netherlands;
3. Department of Internal Medicine, Erasmus Medical Center, Rotterdam, The Netherlands;
4. Department of Cardiology, Radboud University Medical Center, Nijmegen, The Netherlands;

* These authors contributed equally

Ascending aorta measurements

Ascending aortic dimensions were measured at 4, 8 and 12 weeks of age across all groups. While MFS mice exhibit a subtle increase in ascending aortic diameter compared to their WT counterparts at 8 and 12 weeks of age, this difference was not consistently significant. Thus, the ascending aorta does not serve as a suitable indicator for evaluating the treatments effects in *Fbn1*$^{C1041G/+}$ mice.

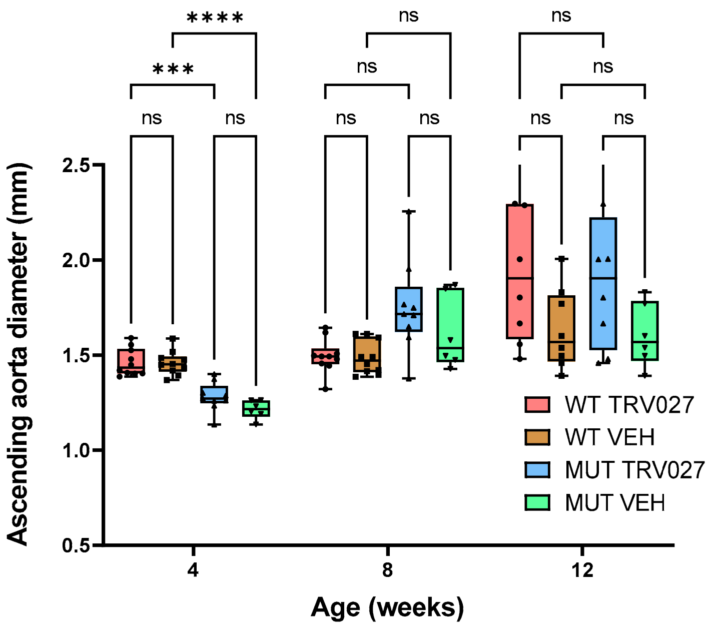

Figure S1. Ascending aorta diameters of mice treated with TRV027 and saline solution. MFS mice show a slight increase of the ascending aortic diameter at 8 and 12 weeks of age, although this increase is not always statistically significant. No differences between MFS mice treated with TRV027 and those given saline solution are observed. Statistical test analysis: one-way ANOVA and Tukey post-test per timepoint. *** p ≤ 0.001, **p ≤ 0.01 and *p ≤ 0.05. VEH: vehicle, WT: Wild-type, MUT: mutant.

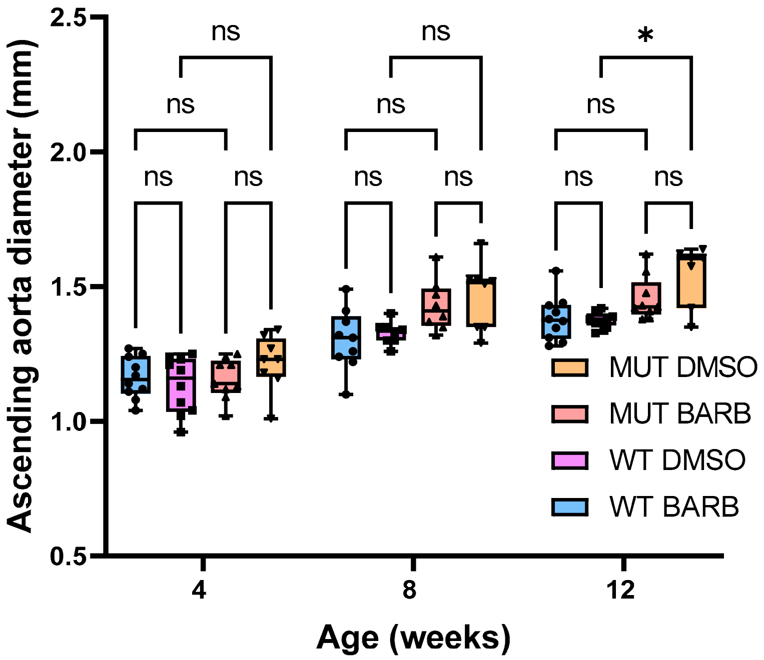

Figure S2. Ascending aorta diameters of mice treated with barbadin + losartan (25 mg/kg/day) and mice treated with losartan (25 mg/kg/day) alone. A slight increase in ascending aortic diameter is observed in MFS mice at 8 and 12 weeks of age, although this increase is not always statistically significant. No significant differences between MFS mice treated with barbadin + losartan (25 mg/kg/day) and mice treated with losartan (25 mg/kg/day) alone are observed. Statistical test analysis: one-way ANOVA and Tukey post-test per timepoint. *** $p \leq 0.001$, **$p \leq 0.01$ and *$p \leq 0.05$. VEH: vehicle, LOS: Losartan, BARB: Barbadin, WT: Wild-type, MUT: mutant.

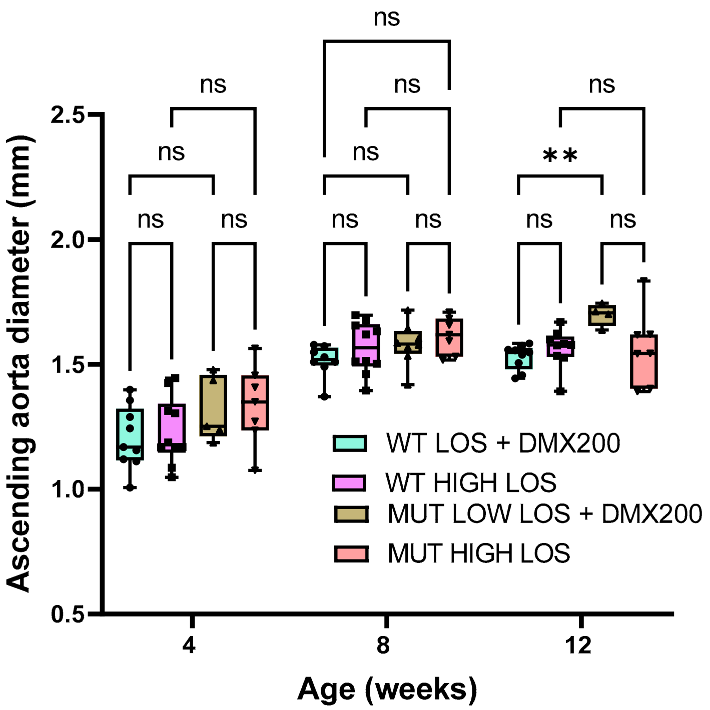

Figure S3. Ascending aorta diameters of mice treated with a high dose of losartan (50 mg/kg/day) and mice treated with DMX200 + losartan (5 mg/kg/day). MFS mice display a subtle increase in ascending aortic diameters at 8 and 12 weeks of age, although this increase is not always significant. MFS mice that were treated with high dose of losartan (50 mg/kg/day) do not exhibit a visually discernible or statistically significant increase compared to their WT counterparts. Statistical test analysis: one-way ANOVA and Tukey post-test per timepoint. *** p ≤ 0.001, **p ≤ 0.01 and *p ≤ 0.05. VEH: vehicle, LOS: Losartan, WT: Wild-type, MUT: mutant.

Weights measurements

Mice weights were recorded prior to performing echocardiography at 4, 8 and 12 weeks of age.

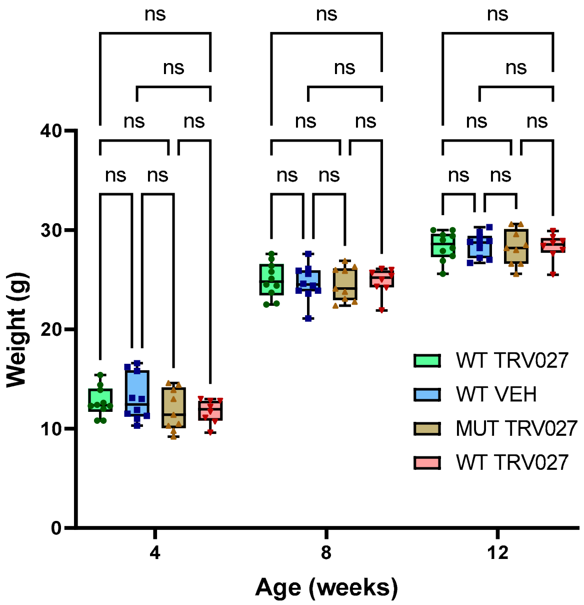

Figure S4. Comparison of the weights from mice treated with TRV027 and saline solution. No significant differences are observed in weight between MFS and WT mice, or between mice treated with TRV027 and saline solution. Statistical test analysis: one-way ANOVA and Tukey post-test per timepoint. *** p ≤ 0.001, **p ≤ 0.01 and *p ≤ 0.05. VEH: vehicle, WT: Wild-type, MUT: mutant.

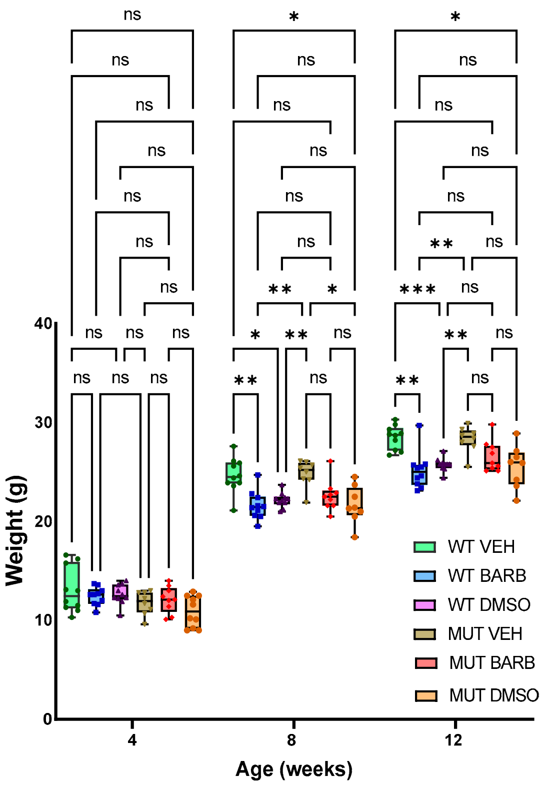

Figure S5. Comparison of the weights between mice treated with barbadin + losartan (25 mg/kg/day), losartan alone (25 mg/kg/day), and with saline solution. Mice that received peritoneal injections of either barbadin or DMSO tend to show lower weights compared to the vehicle group (saline solution). This difference, however, is not consistently significant. Statistical test analysis: one-way ANOVA and Tukey post-test per timepoint. *** $p \leq 0.001$, ** $p \leq 0.01$ and * $p \leq 0.05$. EH: vehicle, LOS: Losartan, BARB: Barbadin, WT: Wild-type, MUT: mutant.

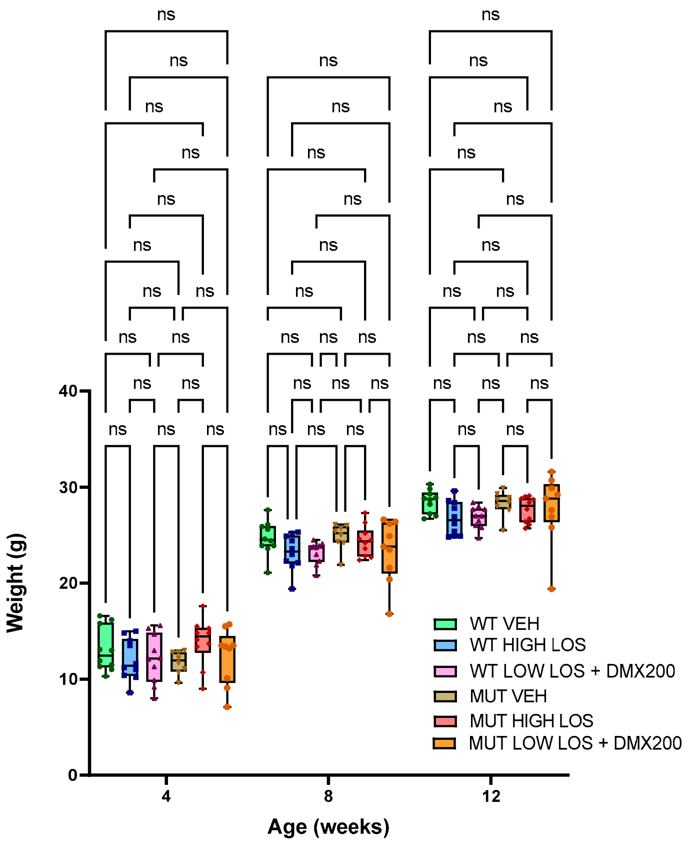

Figure S6. Comparison of the weights between mice treated with DMX200 + losartan (5 mg/kg/day), losartan alone (50 mg/kg/day), and saline solution. No significant differences in weight are observed among mice in these groups. Statistical test analysis: one-way ANOVA and Tukey post-test per timepoint. *** $p \leq 0.001$, **$p \leq 0.01$ and *$p \leq 0.05$. VEH: vehicle, LOS: Losartan, WT: Wild-type, MUT: mutant.

Comparison of weight-normalised aortic root diameters between barbadin, barbadin + losartan and vehicle groups.

Since some differences in weight were observed among mice receiving peritoneal injections of either barbadin or DMSO in comparison to those treated with saline solution (FigS5), we also performed a comparison where diameters were adjusted by weight. This normalization process involves multiplying the average value of each individual mouse by its weight and dividing it by the average weight across all animals. This weight-normalised comparison also showed that the addition of barbadin to losartan treatment does not provide any benefits regarding the aortic root dilatation of MFS mice (Fig.S7).

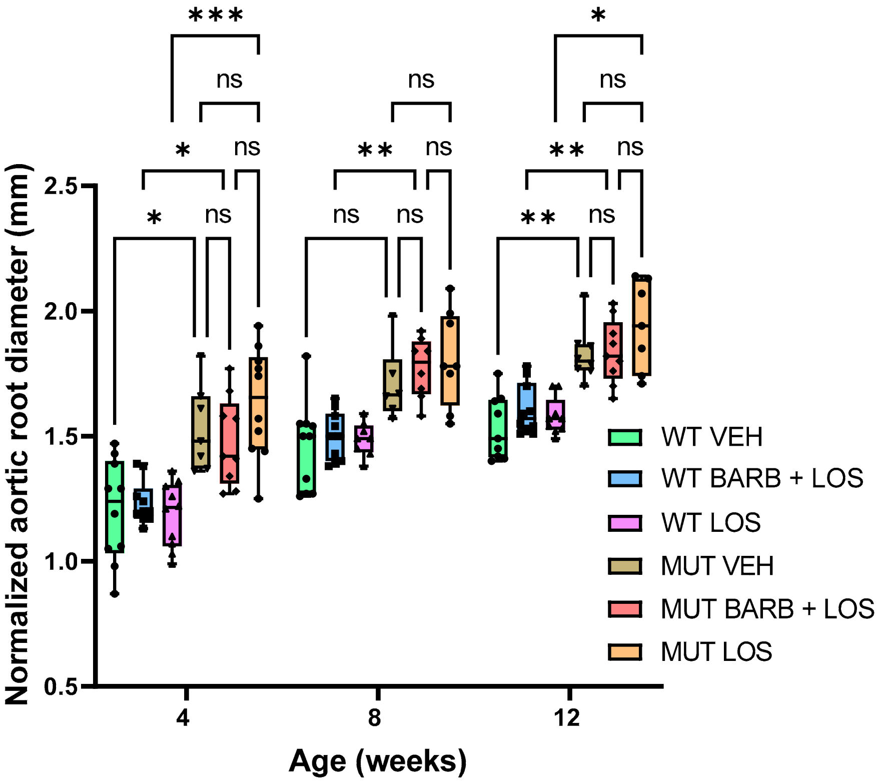

Figure S7. Weight-normalised aortic root diameters of mice treated with barbadin + losartan (25 mg/kg/day) and mice treated with losartan (25 mg/kg/day) alone. Barbadin + losartan treatment does not have any significantly benefit over those treated with losartan alone. Data represented as boxplots. Statistical test analysis: one-way ANOVA and Tukey post-test per timepoint. *** p ≤ 0.001, **p ≤ 0.01 and *p ≤ 0.05. VEH: vehicle, LOS: Losartan, BARB: Barbadin, WT: Wild-type, MUT: mutant.

Statistical parameters

Table S1. Effects sizes, confidence interval and adjusted p-value for the different comparisons of the aortic root diameters between vehicle groups (WT VEH, MUT VEH) and TRV027 groups (WT TRV027, MUT TRV027). Test: one-way ANOVA and Tukey post-test per timepoint.

Tukey's multiple comparisons test	Mean Diff.	95.00% CI of diff.	Adjusted P Value
**4 weeks**			
WT TRV027 vs. WT VEH	0.003933	-0.08173 to 0.08959	0.9992
WT TRV027 vs. MUT TRV027	0.1813	0.08303 to 0.2796	0.0004
WT TRV027 vs. MUT VEH	0.2479	0.1596 to 0.3362	<0.0001
WT VEH vs. MUT TRV027	0.1774	0.08377 to 0.2710	0.0003
WT VEH vs. MUT VEH	0.244	0.1612 to 0.3267	<0.0001
MUT TRV027 vs. MUT VEH	0.06659	-0.02912 to 0.1623	0.2228
**8 weeks**			

WT TRV027 vs. WT VEH	0.008633	-0.1042 to 0.1215	0.9963
WT TRV027 vs. MUT TRV027	-0.257	-0.5200 to 0.005986	0.0558
WT TRV027 vs. MUT VEH	-0.1206	-0.4087 to 0.1675	0.526
WT VEH vs. MUT TRV027	-0.2657	-0.5286 to -0.002717	0.0476
WT VEH vs. MUT VEH	-0.1293	-0.4174 to 0.1589	0.4742
MUT TRV027 vs. MUT VEH	0.1364	-0.1992 to 0.4719	0.6375
**12 weeks**			
WT TRV027 vs. WT VEH	0.3633	-0.2064 to 0.9330	0.2654
WT TRV027 vs. MUT TRV027	0.0475	-0.6472 to 0.7422	0.9971
WT TRV027 vs. MUT VEH	0.3953	-0.1725 to 0.9631	0.2032
WT VEH vs. MUT TRV027	-0.3158	-0.8848 to 0.2532	0.37
WT VEH vs. MUT VEH	0.03199	-0.2693 to 0.3332	0.9886
MUT TRV027 vs. MUT VEH	0.3478	-0.2193 to 0.9148	0.2888

Table S2. Effects sizes, confidence interval and adjusted p-value for the different comparisons of the aortic root diameters between vehicle groups (WT VEH, MUT VEH), barbadin + medium dose losartan (WT BARB + LOS, MUT BARB+ LOS) and medium dose losartan (WT LOS, MUT LOS). Test: one-way ANOVA and Tukey post-test per timepoint.

Tukey's multiple comparisons test	Mean Diff.	95.00% CI of diff.	Adjusted P Value
**4 weeks**			
WT VEH vs. WT LOS	0.061	-0.08458 to 0.2066	0.7438
WT VEH vs. WT BARB + LOS	0.0287	-0.1166 to 0.1740	0.9852
WT VEH vs. MUT VEH	-0.1705	-0.3101 to -0.03085	0.0147
WT VEH vs. MUT LOS	-0.1653	-0.3654 to 0.03482	0.1355
WT VEH vs. MUT BARB + LOS	-0.1692	-0.3123 to -0.02620	0.0171
WT LOS vs. WT BARB + LOS	-0.0323	-0.1318 to 0.06716	0.9009
WT LOS vs. MUT VEH	-0.2315	-0.3180 to -0.1450	<0.0001
WT LOS vs. MUT LOS	-0.2263	-0.4086 to -0.04399	0.0141
WT LOS vs. MUT BARB + LOS	-0.2302	-0.3250 to -0.1355	<0.0001
WT BARB + LOS vs. MUT VEH	-0.1992	-0.2848 to -0.1135	<0.0001
WT BARB + LOS vs. MUT LOS	-0.194	-0.3761 to -0.01184	0.0355
WT BARB + LOS vs. MUT BARB + LOS	-0.1979	-0.2920 to -0.1039	<0.0001
MUT VEH vs. MUT LOS	0.005196	-0.1749 to 0.1853	>0.9999
MUT VEH vs. MUT BARB + LOS	0.001238	-0.07828 to 0.08076	>0.9999
MUT LOS vs. MUT BARB + LOS	-0.003958	-0.1851 to 0.1772	>0.9999
**8 weeks**			
WT VEH vs. WT LOS	0.1261	-0.03069 to 0.2829	0.1406
WT VEH vs. WT BARB + LOS	0.1509	-0.01325 to 0.3151	0.08
WT VEH vs. MUT VEH	-0.2745	-0.4462 to -0.1028	0.0014
WT VEH vs. MUT LOS	-0.1404	-0.3664 to 0.08565	0.3737
WT VEH vs. MUT BARB + LOS	-0.1764	-0.3423 to -0.01047	0.0344
WT LOS vs. WT BARB + LOS	0.02482	-0.06608 to 0.1157	0.9391
WT LOS vs. MUT VEH	-0.4006	-0.5189 to -0.2822	<0.0001

WT LOS vs. MUT LOS	-0.2665	-0.4693 to -0.06370	0.0118
WT LOS vs. MUT BARB + LOS	-0.3025	-0.4006 to -0.2044	<0.0001
WT BARB + LOS vs. MUT VEH	-0.4254	-0.5523 to -0.2986	<0.0001
WT BARB + LOS vs. MUT LOS	-0.2913	-0.4965 to -0.08613	0.0058
WT BARB + LOS vs. MUT BARB + LOS	-0.3273	-0.4412 to -0.2134	<0.0001
MUT VEH vs. MUT LOS	0.1341	-0.07540 to 0.3436	0.3103
MUT VEH vs. MUT BARB + LOS	0.0981	-0.03194 to 0.2281	0.1843
MUT LOS vs. MUT BARB + LOS	-0.036	-0.2422 to 0.1702	0.988
**12 weeks**			
WT VEH vs. WT LOS	0.1102	-0.05105 to 0.2715	0.2701
WT VEH vs. WT BARB + LOS	0.1249	-0.05144 to 0.3012	0.2565
WT VEH vs. MUT VEH	-0.3027	-0.4678 to -0.1376	0.0004
WT VEH vs. MUT LOS	-0.2145	-0.4620 to 0.03292	0.1034
WT VEH vs. MUT BARB + LOS	-0.192	-0.3518 to -0.03224	0.016
WT LOS vs. WT BARB + LOS	0.01467	-0.1207 to 0.1500	0.9991
WT LOS vs. MUT VEH	-0.413	-0.5282 to -0.2978	<0.0001
WT LOS vs. MUT LOS	-0.3248	-0.5596 to -0.08994	0.0087
WT LOS vs. MUT BARB + LOS	-0.3022	-0.4048 to -0.1996	<0.0001
WT BARB + LOS vs. MUT VEH	-0.4276	-0.5686 to -0.2867	<0.0001
WT BARB + LOS vs. MUT LOS	-0.3394	-0.5788 to -0.1001	0.0058
WT BARB + LOS vs. MUT BARB + LOS	-0.3169	-0.4499 to -0.1838	<0.0001
MUT VEH vs. MUT LOS	0.0882	-0.1475 to 0.3239	0.7513
MUT VEH vs. MUT BARB + LOS	0.1107	-0.001363 to 0.2228	0.0537
MUT LOS vs. MUT BARB + LOS	0.02254	-0.2121 to 0.2572	0.9989

Table S3. Effects sizes, confidence interval and adjusted p-value for the different comparisons of the aortic root diameters between vehicle groups (WT VEH, MUT VEH), DMX200 + low dose losartan (WT low LOS + DMX200, VEH low LOS + DMX200) and high dose of losartan (WT high LOS, MUT high LOS). Test: one-way ANOVA and Tukey post-test per timepoint.

Tukey's multiple comparisons test	Mean Diff.	95.00% CI of diff.	Adjusted P Value
**4 weeks**			
WT VEH vs. WT high LOS	-0.0208	-0.1764 to 0.1348	0.9978
WT VEH vs. WT low LOS + DMX200	-0.002511	-0.1627 to 0.1577	>0.9999
WT VEH vs. MUT VEH	-0.177	-0.3435 to -0.01055	0.0338
WT VEH vs. MUT high LOS	-0.1691	-0.3486 to 0.01042	0.0716
WT VEH vs. MUT low LOS + DMX200	-0.2577	-0.4964 to -0.01897	0.0323
WT high LOS vs. WT low LOS + DMX200	0.01829	-0.1187 to 0.1553	0.9978
WT high LOS vs. MUT VEH	-0.1562	-0.3018 to -0.01060	0.0322
WT high LOS vs. MUT high LOS	-0.1483	-0.3098 to 0.01322	0.0818
WT high LOS vs. MUT low LOS + DMX200	-0.2369	-0.4684 to -0.005401	0.0444
WT low LOS + DMX200 vs. MUT VEH	-0.1745	-0.3253 to -0.02369	0.0193
WT low LOS + DMX200 vs. MUT high LOS	-0.1666	-0.3324 to -0.0007647	0.0486

WT low LOS + DMX200 vs. MUT low LOS + DMX200	-0.2552	-0.4882 to -0.02218	0.0306
MUT VEH vs. MUT high LOS	0.007925	-0.1638 to 0.1796	>0.9999
MUT VEH vs. MUT low LOS + DMX200	-0.08066	-0.3161 to 0.1547	0.8298
MUT high LOS vs. MUT low LOS + DMX200	-0.08859	-0.3296 to 0.1524	0.808
**8 weeks**			
WT VEH vs. WT high LOS	0.0982	-0.06969 to 0.2661	0.4327
WT VEH vs. WT low LOS + DMX200	0.06262	-0.1154 to 0.2407	0.8606
WT VEH vs. MUT VEH	-0.2029	-0.3671 to -0.03871	0.013
WT VEH vs. MUT high LOS	-0.03493	-0.2540 to 0.1842	0.9949
WT VEH vs. MUT low LOS + DMX200	-0.2729	-0.5217 to -0.02413	0.0295
WT high LOS vs. WT low LOS + DMX200	-0.03558	-0.1688 to 0.09763	0.9497
WT high LOS vs. MUT VEH	-0.3011	-0.4065 to -0.1957	<0.0001
WT high LOS vs. MUT high LOS	-0.1331	-0.3266 to 0.06033	0.2591
WT high LOS vs. MUT low LOS + DMX200	-0.3711	-0.6084 to -0.1339	0.0049
WT low LOS + DMX200 vs. MUT VEH	-0.2655	-0.3931 to -0.1379	0.0001
WT low LOS + DMX200 vs. MUT high LOS	-0.09756	-0.2984 to 0.1033	0.6113
WT low LOS + DMX200 vs. MUT low LOS + DMX200	-0.3356	-0.5746 to -0.09652	0.0076
MUT VEH vs. MUT high LOS	0.168	-0.02310 to 0.3590	0.0951
MUT VEH vs. MUT low LOS + DMX200	-0.07005	-0.3085 to 0.1684	0.8445
MUT high LOS vs. MUT low LOS + DMX200	-0.238	-0.4981 to 0.02213	0.08
**12 weeks**			
WT VEH vs. WT high LOS	0.1152	-0.02442 to 0.2548	0.1396
WT VEH vs. WT low LOS + DMX200	0.03887	-0.1286 to 0.2064	0.9732
WT VEH vs. MUT VEH	-0.2644	-0.4143 to -0.1145	0.0005
WT VEH vs. MUT high LOS	-0.0348	-0.2025 to 0.1329	0.9842
WT VEH vs. MUT low LOS + DMX200	-0.1839	-0.3718 to 0.003888	0.0563
WT high LOS vs. WT low LOS + DMX200	-0.07633	-0.2271 to 0.07440	0.5762
WT high LOS vs. MUT VEH	-0.3796	-0.5083 to -0.2509	<0.0001
WT high LOS vs. MUT high LOS	-0.15	-0.3006 to 0.0005780	0.0512
WT high LOS vs. MUT low LOS + DMX200	-0.2991	-0.4757 to -0.1226	0.0016
WT low LOS + DMX200 vs. MUT VEH	-0.3032	-0.4628 to -0.1437	0.0003
WT low LOS + DMX200 vs. MUT high LOS	-0.07367	-0.2491 to 0.1017	0.7578
WT low LOS + DMX200 vs. MUT low LOS + DMX200	-0.2228	-0.4166 to -0.02899	0.0209
MUT VEH vs. MUT high LOS	0.2296	0.07012 to 0.3890	0.0033
MUT VEH vs. MUT low LOS + DMX200	0.08043	-0.1020 to 0.2629	0.6627
MUT high LOS vs. MUT low LOS + DMX200	-0.1491	-0.3430 to 0.04476	0.1809

Table S4. Descriptive statistics (mean and standard deviation) for the different groups at the three timepoints (4,8, 12 weeks).

Group	Statistics	4 weeks	8 weeks	12 weeks
WT TRV027	Mean	1.29	1.62	1.70
	SD	0.08	0.10	0.09
MUT TRV027	Mean	1.51	1.89	2.03
	SD	0.09	0.07	0.12
WT VEH	Mean	1.33	1.62	1.69
	SD	0.12	0.14	0.11
MUT VEH	Mean	1.50	1.82	1.96
	SD	0.10	0.05	0.08
WT medium LOS	Mean	1.22	1.44	1.53
	SD	0.07	0.04	0.07
MUT medium LOS	Mean	1.45	1.70	1.85
	SD	0.13	0.15	0.16
WT BARB + medium LOS	Mean	1.26	1.41	1.51
	SD	0.07	0.08	0.11
MUT BARB + medium LOS	Mean	1.45	1.74	1.83
	SD	0.06	0.07	0.06
WT high LOS	Mean	1.35	1.52	1.58
	SD	0.09	0.08	0.08
MUT high LOS	Mean	1.49	1.66	1.73
	SD	0.13	0.15	0.12
WT low LOS + DMX200	Mean	1.33	1.56	1.65
	SD	0.09	0.10	0.12
MUT low LOS + DMX200	Mean	1.58	1.89	1.88
	SD	0.15	0.14	0.12
